# The Symptom and Genetic Diversity of Cassava Brown Streak Viruses Infecting Cassava in East Africa

**DOI:** 10.1155/2012/795697

**Published:** 2012-02-21

**Authors:** I. U. Mohammed, M. M. Abarshi, B. Muli, R. J. Hillocks, M. N. Maruthi

**Affiliations:** ^1^Natural Resources Institute, University of Greenwich, Chatham Maritime, Kent ME4 4TB, UK; ^2^Food Crops Programme, Kenya Agricultural Research Institute, P. O. Box 16-80109, Mtwapa, Kenya

## Abstract

The genetic and symptom diversity of six virus isolates causing cassava brown streak disease (CBSD) in the endemic (Kenya, Mozambique, and Tanzania) and the recently affected epidemic areas (Uganda) of eastern Africa was studied. Five cassava varieties; Albert, Colombian, Ebwanateraka, TMS60444 (all susceptible) and Kiroba (tolerant) were graft inoculated with each isolate. Based on a number of parameters including the severity of leaf and root symptoms, and the extent of virus transmission by grafting, the viruses were classified as either severe or relatively mild. These results were further confirmed by the mechanical inoculation of 13 herbaceous hosts in which the virulent isolates caused plant death in *Nicotiana clevelandii* and *N. benthamiana* whereas the milder isolates did not. Phylogenetic analysis of complete coat protein gene sequences of these isolates together with sequences obtained from 14 other field-collected samples from Kenya and Zanzibar, and reference sequences grouped them into two distinct clusters, representing the two species of cassava brown streak viruses. Put together, these results did not suggest the association of a hypervirulent form of the virus with the current CBSD epidemic in Uganda. Identification of the severe and milder isolates, however, has further implications for disease management and quarantine requirements.

## 1. Introduction

Cassava brown streak disease (CBSD) is endemic in areas along the Indian Ocean coast of eastern Africa, from the northeastern border of Kenya across the Tanzanian border down as far as the Zambezi River in Mozambique, and it was widespread around the shore of Lake Malawi. In the endemic areas, CBSD was confined to altitudes below 1,000 metres above sea level [1–3]. More recently, CBSD has been reported at midaltitude levels (1200–1500 meters above sea levels) in Democratic Republic Congo [4], Uganda [5], and the Lake zone areas of Tanzania [6, 7], which were not considered to be at risk by the disease previously. This is a serious concern because the disease incidences of up to 100% were recorded [8], and in sensitive varieties the disease causes rotting of tubers, reducing both the quality and quantity of tubers available for consumption [1, 2, 9]. A moderate infection by CBSD (10–30% damage to root surface area) decreases the market value of cassava tubers drastically by 90%, fetching under US $5 per tonne, as opposed to $55 for fresh healthy cassava root [10]. Severely diseased roots are completely destroyed and unfit for market or family use. Recent estimates indicate that CBSD causes economic losses of up to $100 million annually to the African farmer [11] and these are probably an underestimate, as the disease has since spread into new areas [5, 7]. The disease is now considered to be the most important cause of food insecurity in the coastal and lake zone areas of eastern Africa.

Based on complete genome sequences, CBSD is known to be caused by two distinct virus species. The coastal endemic virus is referred to as *Cassava brown streak virus* (CBSV), and the highland epidemic virus as *Cassava brown streak Uganda virus* (CBSUV). Both species belong to the genus *Ipomovirus*, family *Potyviridae* [12–16], and are transmitted by the whiteflies (*Bemisia tabaci* Gennadius) [17, 18].

The prominent symptoms of CBSD appear on leaves with varying patterns of chlorosis which enabled Nichols [2] to distinguish two types of CBSD isolates. Leaf chlorosis appear in a feathery pattern, first along the margins of the secondary veins, later affecting tertiary veins and may develop into chlorotic blotches. Alternatively, the chlorosis may not be clearly associated with the veins but appear in roughly circular patches between the main veins. There is considerable variation in the expression of foliar symptoms depending on variety, growing conditions (temperature, rainfall, and altitude), age of the plant, and the virus isolate involved in causing the symptoms. Some cultivars show marked foliar symptoms but without or delayed root symptoms (example var. Kiroba), and *vice versa*. Symptoms of the disease are more difficult to recognize in older plants as the lower leaves with prominent symptoms senesce and fall off. New leaves produced from these plants often do not show symptoms, especially at high temperatures. Symptoms can be also transient when a period of active growth produces symptom-free tissues [19]. However, it is difficult to interpret these observations precisely because they have been made in field situations with varying agroclimatic conditions, on cassava varieties with differing virus resistance levels and crop age, and possibly infected with different virus strains, which on their own or in combinations affected symptom development. The main objectives of this study were, therefore, to study symptom diversity of CBSV and CBSUV isolates under uniform environmental conditions, and to identify whether the CBSD epidemic was associated with a severe form of the virus. Accordingly, six CBSD isolates from endemic (Mozambique, Kenya, Tanzania, and Zanzibar) and epidemic areas (Uganda) were each inoculated to five cassava varieties with varying levels of virus tolerance. The effect of each virus on plant growth and symptom development were recorded under standard conditions in a glasshouse. Symptom diversity was also investigated by inoculating 13 species of experimental host-plants. The genetic diversity of the virus isolates was estimated by cloning and sequencing complete coat protein (CP) genes.

## 2. Materials and Methods

### 2.1. Cassava Varieties and CBSD Isolates

Stem cuttings of five disease-free cassava varieties Ebwanateraka (collected from Uganda) and Albert and Kiroba (both collected from Tanzania) were collected from farmer's fields. Variety Colombian was obtained from the University of Bristol, UK, and TMS60444 from the International Laboratory for Tropical Agricultural Biotechnology (ILTAB), St. Louis, USA. Plants were grown at 28 ± 5°C, 50–60% relative humidity in the quarantine glasshouse at the Natural Resources Institute (NRI), UK, and observed for cassava mosaic disease (CMD) and CBSD symptoms. Plants were virus-indexed using reverse transcription polymerase chain reaction (RT-PCR) tests and the absence of two cassava brown streak viruses (CBSVs) using primers designed in this study (see below) as well as for cassava mosaic begomoviruses (CMB) [20, 21]. Symptomless plants in which no virus was detected was further cultivated through the micropropagation of nodal buds using tissue culture techniques [22]. PCR was used on tissue-cultured plants to further confirm the absence of CBSVs and CMBs. The resulting virus-free plants were used in subsequent virus inoculation experiments. 

**Figure 1:**
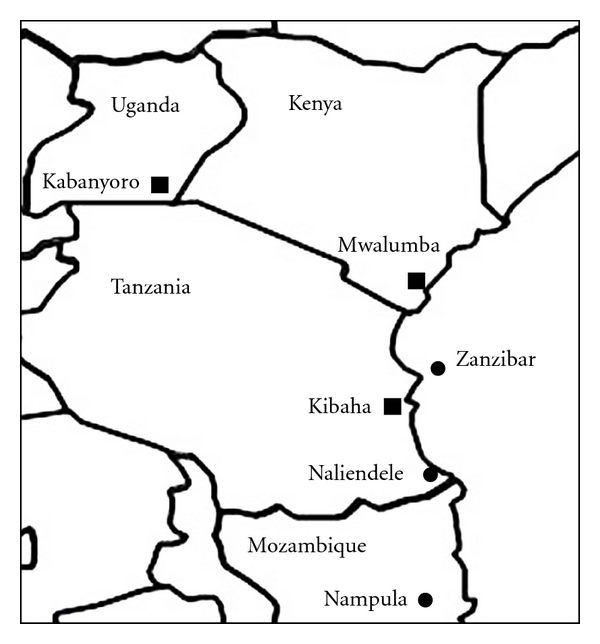
A sketch map of eastern Africa showing the collection sites of CBSV (circles) and CBSUV (squares) isolates used in symptom diversity studies.

 The six CBSD virus isolates used in this study were collected as stem cuttings of unknown cassava varieties in farmer's fields (Table 1, Figure 1) from disease endemic areas in Nampula, Mozambique (CBSV-[MZ:Nam1-1:07]); Naliendele, Tanzania (CBSV-[TZ:Nal3-1:07]); Zanzibar, Tanzania (CBSV-[TZ:Zan6-2:08]); Mwalumba, Kenya (CBSUV-[KE:Mwa16-2:08]); Kibaha, Tanzania (CBSUV-[TZ:Kib10-2:03]), and from the epidemic area of Kabanyoro, Uganda (CBSUV-[UG:Kab4-3:07]) [23, 24]. The identity of the viruses was confirmed using RT-PCR, cloning, and sequencing of the complete CP genes (see below).

### 2.2. Graft Inoculation of Virus Isolates and Recording Symptom Severity

The six CBSD isolates were grafted onto two-month-old healthy cassava plants of five cassava varieties: Ebwanateraka, Albert, Kiroba, Colombian, and TMS60444. Plants were kept in constant environment at 28 ± 5°C and 50–60% relative humidity for symptom development. Various parameters were recorded at weekly intervals for determining CBSD symptom severity on cassava leaves and roots, herbaceous hosts (see below), the rate of graft-transmission, the sprouting of the infected cassava cuttings and virus titres in infected plants. Ten cuttings were made for each virus-variety combination (6 viruses × 5 varieties = 30 treatments) which resulted in a total of 300 cuttings. Numbers of cuttings that sprouted was recorded to measure the effect of CBSD on sprouting young cuttings.

 Leaf symptoms severity was scored on 3-month-old plants using a five point scale where 1 = no visible CBSD symptoms, 2 = mild foliar symptoms on some leaves, 3 = pronounced foliar symptoms but no die-back, 4 = pronounced foliar symptoms which might include slight die-back of terminal branches, and 5 = severe foliar symptoms and plant die-back [9, 25]. Root symptoms were recorded about 18 months after planting by horizontally cutting the tubers for every 1-2 cm.

### 2.3. Sap-Inoculation of Herbaceous Host-Plants

Thirteen herbaceous species/varieties were tested for their response to CBSVs through sap-inoculations (Table 2). The 0.06 M potassium phosphate buffer was prepared (80.2 mL of 0.6 M K_2_HPO_4_ + 19.8 mL of 0.6 M KH_2_PO_4_ + 900 mL of SDW) and pH was adjusted to 7.4 and autoclaved. For each isolate, a cassava leaf showing clear CBSD symptom was collected and ground separately in ~20 mL of the potassium phosphate buffer using pestle and mortar. The leaf debris was separated from the sap by squeezing through sterile muslin cloth. Fully-open young leaves of herbaceous plants were sprinkled with fine 600 mesh carborundum powder and the plant sap was applied gently using a cotton wool pad stroking from petiole to the leaf tip. Virus inoculated leaves were rinsed thoroughly using a jet of water 10 min after the application of sap and the plants were kept for symptom development. Plants inoculated with buffer alone served as controls.

### 2.4. Detection of CBSVs and Estimation of Virus Titres

 Total nucleic acids were extracted separately from cassava leaves infected with each virus isolate using the modified cetyl trimethyl ammonium bromide (CTAB) method [21, 26, 27]. For the purposes of designing virus-specific primers, the 12 complete sequences of CBSVs that were available in gene bank database European Molecular Biology Laboratory (EMBL) were aligned and the primers were designed to the most conserved regions in the 3′ terminal region of the genome. A single forward degenerate primer CBSVF2 (5′ GGR CCA TAC ATY AAR TGG TT 3′) common to CBSVs was designed in the middle of the conserved HAM1h protein about 250 bases upstream of the 5′ end of CP. The two reverse primers CBSVR7 (5′ CCC TTT GCA AAR CTR AAA TARC 3′) and CBSVR8 (5′ CCA TTR TCT YTC CAM ADC TTC 3′) specific to CBSUV and CBSV, respectively, were designed in the conserved regions of 3′ untranslated region (UTR). Viral cDNAs were prepared from two samples of the graft-inoculated cassava plants for each isolate using the OligodT primer and the RT-PCR was carried out as described by Abarshi et al. [27]. For estimating relative concentrations of virus particles, cDNAs were diluted serially: 10^−1^, 10^−2^, 10^−3^, 10^−4^ and 10^−5^, and the viral genomes amplified using RT-PCR.

### 2.5. Cloning and Sequencing of the Virus Coat Protein Gene

 The CBSVF2 was used in combination with another degenerate primer CBSVR1 (5′ AAY ARA AAG GAT ATG GAG AAA G 3′) to amplify the complete CP of CBSVs following the protocols of Abarshi et al. [27]. CBSVR1 was designed to the conserved region of 3′ UTR and together with CBSVF2, these primers amplified approximately 1,600 bp fragment encompassing the partial HAM1 gene, partial 3′ UTR, and complete CP of CBSVs. The RT-PCR amplicons obtained were cloned into pGEMT Easy vector (Promega, UK) and sequenced. For each sample two clones were sequenced in both directions where possible. Sequences were edited and aligned using the software package MEGA4 [28]. BLAST search analysis was carried out to confirm the identity of the sequences. Maximum parsimony analysis and heuristic search were used to generate the most parsimonious phylogenetic tree. The reliability of the tree was estimated by performing 1,000 bootstrap repetitions. The CP sequences of all CBSD isolates were compared with those reference sequences obtained from the EMBL database (accession numbers: FN434109, FJ185044, FN433931, FN433933, FN433932, FJ039520, FN433930, FN434436 and GQ329864).

### 2.6. CBSV Genetic Diversity in Field Samples

In order to correlate symptom variation observed with the genetic diversity of the viruses in the field, a mini survey was carried out in October 2008 for CBSD in the coastal regions of Kenya north and south of Mombasa city [23]. The survey extended about 120 km to the south towards the Tanzanian border along the main road A14, and to the north of Mombasa along B8 up to the town of Kilifi. Cassava leaves showing CBSD symptoms were collected in farmers' fields neighbouring the highways at every 15–20 km intervals. Five CBSD affected leaf samples were also collected for analysis from farmer fields on the Island of Zanzibar during the same period. Genomes of CBSVs were amplified from field-collected samples using CBSVF2 and CBSVR1 primers and the PCR products were cloned and sequenced as above.

## 3. Results

### 3.1. Parameters Measured to Estimate CBSD Symptom Severity on Cassava

**Efficiency of Graft Transmission**

Of the 30 plants graft inoculated for each virus and variety combination, all plants inoculated with CBSV-[TZ:Nal3-1:07] and CBSV-[MZ:Nam1-1:07] resulted in infections (Table 3). The efficiency of infection varied for the remaining isolates as only 60% of the plants became infected with the epidemic isolate CBSUV-[UG:Kab4-3:07]. No significant differences were observed amongst the varieties in the rate of CBSD infection, which varied from 77 to 87%.

**Sprouting of the Infected Cuttings**

Amongst the isolates, maximum number of cuttings were sprouted from the epidemic isolate CBSUV-[UG:Kab4-3:07] (96%) and the least number of cuttings from CBSV-[TZ:Nal3-1:07] (74%) (Table 4). No significant differences were observed amongst the varieties except for TMS60444 from which only 67% of the cuttings were sprouted.

**Leaf Symptom Severity Scores**

**Figure 2:**
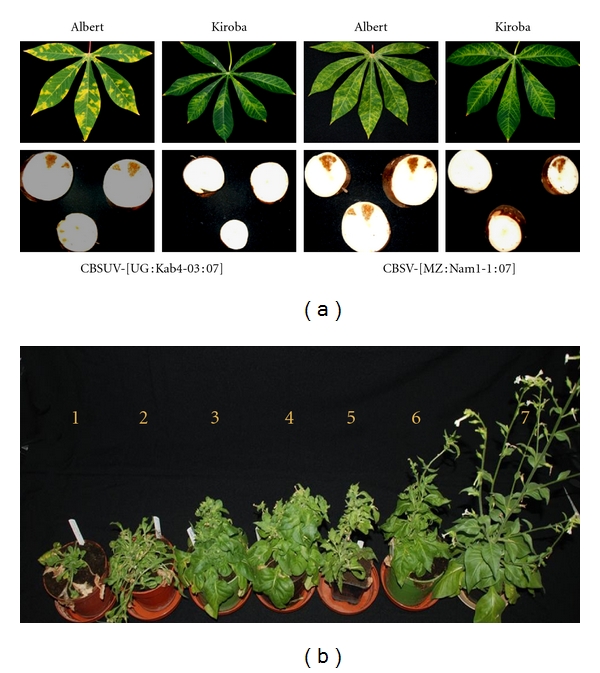
(a) Typical leaf and root symptoms expressed by the CBSUV-[UG:Kab4-3:07] and CBSV-[MZ:Nam1-1:07] in varieties Albert and Kiroba. (b) Typical symptoms observed on *N. clevelandii* plants 5-6 weeks after inoculated with sap extracted from infected cassava plants. 1 = CBSV-[MZ:Nam1-1:07]; 2 = CBSV-[TZ:Nal3-1:07]; 3 = CBSV-[TZ:Kib10-2:03]; 4 = CBSV-[TZ:Zan6-2:08]; 5 = CBSUV-[KE:Mwa16-2:08]; 6 = CBSUV-[UG: Kab4-3:07]; 7 = healthy control plant.

Mean maximum leaf symptom severity score of 3.8 was recorded for CBSV-[MZ:Nam1-1:07] and the mean minimum score of 1.9 for CBSUV-[UG:Kab4-3:07] (Table 5, Figure 2). The symptom severity score for each variety varied. When a multiple comparison ANOVA was carried out, significantly high differences (*P* < 0.05) were observed for virus-variety interactions on symptom score (data not shown).

**Relative Virus Concentrations**

In a serial dilution of viral cDNA from 10^−1^ to 10^−5^ folds, virus was detectable at 10^−5^ dilutions only from CBSV-[MZ:Nam1-1:07] and CBSV-[TZ:Nal3-1:07] isolates. For the remaining four isolates virus was not detectable at 10^−3^ or greater dilutions.

**Root Symptoms**

Typical necrosis and dry rotting of infected tubers was recorded for all virus-variety combinations except for Kiroba infected with CBSUV-[UG:Kab4-03:07], which did not produce root symptoms (Figure 2).

### 3.2. CBSD Symptom Phenotype on Cassava

CBSD symptoms were variable but two recognisable patterns emerged, which are associated with the virus species involved.

**CBSUV Symptom Pattern**

Initial symptoms of plants infected with CBSUV isolates appeared as faint yellow spots on the affected leaves which later developed into bright yellow patches of usually irregular to occasionally circular shape. The yellow patches were vividly defined especially in susceptible varieties (e.g., Albert, Figure 2). The symptoms were not always associated with veins and not uniformly distributed throughout the leaflet leaving some parts of the leaf unaffected. As the symptoms developed further, most of the symptomatic leaf turned bright yellow while some areas remained green.

**CBSV Symptom Pattern**

Initial symptoms of plants infected with CBSV isolates appeared as faint yellow streaks usually along the tertiary veins which later developed into severe chlorosis and feathery yellowing extending to secondary and primary veins. The yellowing of veins was mostly even, spreading uniformly throughout the affected leaf which unlike CBSUV, symptoms did not develop into concentric bright yellow patches (Figure 2). Necrotic spots were seen on sensing leaves which also appeared completely yellow before leaf fall.

### 3.3. CBSD Symptom Severity on Herbaceous Hosts

All six CBSD isolates infected *Datura stramonium*, *Nicotiana benthamiana, N. clevelandii*, *N. glutinosa, N. tabacum nn, N. tabacum NN, *and* N. rustica* with varying rates of infection (Table 2). All plants of *N. clevelandii* were infected with each isolate. Most but not all plants of *N. tabacum *nn,* N. tabacum *NN, and* N. rustica* were also infected with each isolate. Time taken for first symptom expression on these hosts varied for each isolate and it depended on the plant species infected. Amongst the isolates, CBSV-[MZ:Nam1-1:07] produced symptoms on all hosts within a week of inoculation, which is closely followed by CBSV-[TZ:Nal3-1:07]. Symptom expression ranged from week 1–4 for the remaining five isolates (data not shown). Of the plant species, *N. clevelandii* was most susceptible, showing symptoms on all plants between weeks 1 to 3.

Symptom severity on herbaceous plants varied especially on *N. clevelandii* and *N. benthamiana*. Plants infected with CBSV-[TZ:Nal3-1:07] and CBSV-[MZ:Nam1-1:07] were severely stunted and subsequently wilted by developing leaf necrosis (Figure 2(b)). Most of these plants died usually within four weeks after virus inoculation. Plants infected with the remaining isolates developed various patterns of chlorosis, vein clearing, leaf malformation, and stunting but not necrosis or death. Symptoms on other hosts also varied but in general included leaf chlorosis, mosaic, and mottling. Local lesions were seen on *N. tabacum *nn, chlorosis/mosaic patterns in* N. tabacum *NN, and vein clearing in *N. benthamiana* by all the isolates.

### 3.4. Detection of CBSVs and Virus Diversity

**Figure 3:**
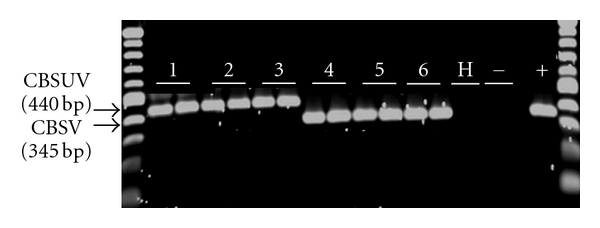
RT-PCR amplification of CBSUV and CBSV genomes using newly designed primers (CBSVF2 in combination with CBSVR7 and CBSVR8). 1 = CBSUV-[UG:Kab4-3:07], 2 = CBSUV-[TZ:Kib10-2:03], 3 = CBSUV-[KE:Mwa16-2:08], 4 = CBSV-[TZ:Zan6-2:08], 5 = CBSV-[MZ:Nam1-1:07], 6 = CBSV-[TZ:Nal3-1:07], H = RNA extraction from a CBSD-free plant, − = negative water control, and + = a known CBSV RNA control from previous sample preparations. The size ladder at each border of the gel is the 100 bp molecular weight markers (New England Biolabs, UK).

All six CBSD isolates were detected by RT-PCR using novel primers. CBSV and CBSUV were distinguished using virus-specific primers; CBSVF2 & CBSVR7 and CBSVF2 and CBSUVR8, which specifically amplified CBSV (345 bp) and CBSUV (440 bp), respectively (Figure 3). No amplifications were obtained from RNA extracted from virus-free plants (healthy). PCR products were sequenced to confirm the specificity of the primers to respective viruses (data shown). CBSVs were detected in all five Zanzibar samples from Tanzania and all but two (Mwatundo and Mwajambo) Kenyan samples. The amplified products contained ~1600 nucleotide sequences upon sequencing. Clones from most samples yielded unique consensus sequences, and those with more than one unique sequence are shown in Table 1 (e.g., three distinct sequences were obtained from Kilifi, and two each from Shirazi and Kikonde).

**Figure 4:**
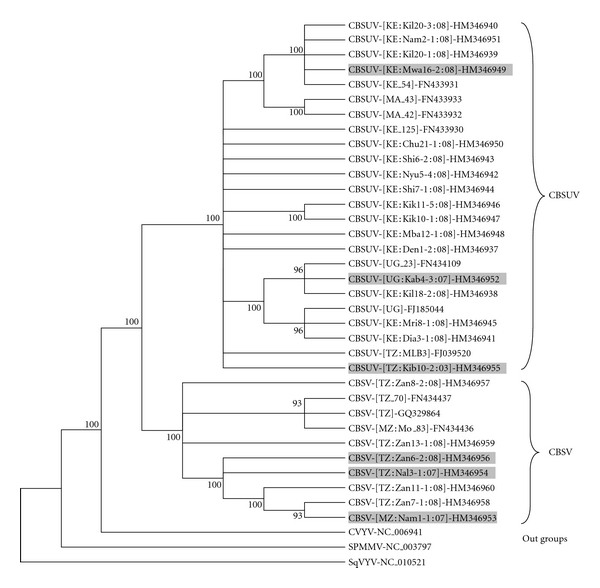
The most parsimonious tree showing the relationship between the two cassava brown streak viruses based on coat protein gene amino acid sequences. The isolates for which symptom diversity was studied are shaded in grey.

The deduced amino acid (aa) sequences for the complete CP of CBSV and CBSUV isolates consisted of 378 and 367 bases, respectively. These were used to estimate their genetic relationships together with the reference sequences of CBSV, CBSUV, *cucumber vein yellowing virus* (CVYV), *squash vein yellowing virus* (SqVYV), and *sweet potato mild mottle virus* (SPMMV). The most parsimony analysis grouped the CP aa sequences into two major clusters: CBSV and CBSUV (Figure 4). Amongst the field-collected samples, all Kenyan sequences belonged to the CBSUV group and all Zanzibar sequences grouped with CBSV. Members within each group were conserved with an average aa similarity of 95.8% for CBSV group, and a 96.6% similarity for CBSUV group. The two groups were similar to each other by only 80.3%. The epidemic isolate CBSUV-[UG:Kab4-03:07] was highly similar (CP aa similarities between 96.9% to 99.2%) to the previously described isolates from Uganda, Kenya, and Malawi.

## 4. Discussion

Until recently, research on CBSD diversity/severity has largely been restricted to observations in the field on cassava plants of different age, genetic makeup, and grown in different agroecological zones with varying environmental conditions and possibly infected with different virus strains, all of which can independently or in combination influence symptom development. This made the comparison of the field observations between the various studies particularly difficult and the question of whether a severe form of CBSD is associated with the latest epidemic in Uganda has remained unanswered. Inoculation of herbaceous host plants by various researchers provided somewhat uniform conditions for symptom diversity studies [29] but until recently no such comparison has been made with isolates from the coastal endemic and inland epidemic areas involving the two different species of CBSVs [15, 16]. It was particularly difficult to conclude whether the severe CBSD symptoms observed in the fields of coastal Mozambique and Tanzania [9], for example, or the relatively milder leaf symptoms seen in Uganda (severity score of 2.0, [5]) were due to the effect of virus isolate or the tolerance/susceptibility of the cassava varieties being grown in those regions. In order to answer these questions, experiments were carried out in controlled environmental conditions in a glasshouse using a standard range of CBSD isolates from both the endemic and epidemic regions to determine the virulence of the isolates. This was particularly relevant to understand if the new outbreaks of CBSD at high altitudes in Uganda and the lake zone areas of Tanzania were due to the prevalence of a severe form of the virus, similar to those observed during the course of the CMD pandemic in Uganda in the early 1990s.

In order to investigate this, a number of parameters were used to assess the severity levels between one epidemic and five endemic CBSD isolates including the symptoms on leaf and roots of five infected cassava varieties, the effect of virus on sprouting of cassava stem cuttings, the rate of graft transmission, and virus titres in infected leaves as well as symptom severity on herbaceous host plants. Amongst the isolates examined, the endemic isolates CBSV-[MZ:Nam1-1:07] and CBSV-[TZ:Nal3-1:07] produced the most severe symptoms with mean symptom severity scores of 3.7-3.8 on a five-point scale [9]. In comparison, the epidemic CBSUV-[UG:Kab4-3:07] isolate produced relatively mild symptoms with a mean leaf severity score of 1.9. These differences were further confirmed upon the inspection of root symptoms in which CBSV-[MZ:Nam1-1:07] and CBSV-[TZ:Nal3-1:07] infections resulted in root necrosis in all five cassava varieties tested including the tolerant variety Kiroba, however, this was not by CBSUV-[UG:Kab4-3:07] (Figure 2). The severity of CBSVs can also be estimated by their ability to affect the young growing buds of infected cassava plants [2, 30]. Using these earlier observations as cues, the differences in the severity levels of the epidemic and endemic isolates were further demonstrated when a significantly higher number of cuttings failed to sprout from the severe endemic isolates compared to the milder epidemic isolate. Between 22 and 26% of the cuttings failed to sprout when infected with CBSV-[MZ:Nam1-1:07] or CBSV-[TZ:Nal3-1:07] while only 4% of the cuttings were similarly affected by the infection of CBSUV-[UG:Kab4-3:07] (Table 4). These observations were further supported by the higher rates of virus transmission by grafting of the endemic severe isolates which is probably due to high virus titre (about 1000-times higher virus titre in the two severe endemic isolates CBSV-[MZ:Nam1-1:07] or CBSV-[TZ:Nal3-1:07] compared to the epidemic isolate CBSUV-[UG:Kab4-3:07]). A notable difference observed between this and earlier studies, however, is the infection of the cassava variety Albert by all isolates of this study. In graft-inoculation experiments, Winter et al. [16] failed to infect Albert by the CBSD isolates from Kenya, Uganda, and Malawi. While the differences between these two similar studies could not be explained at this stage, these results nonetheless have great implications for developing disease management strategies since Albert once considered resistant to CBSD in Kenya, Uganda and Malawi has now been proven susceptible. In southern Tanzania, growing of Albert has been largely abandoned due to its susceptibility to the disease (R. J. Hillocks, unpublished).

The differences in symptoms were also observed on infected herbaceous hosts. Compared to the previously reported *N. benthamiana* [15, 16], *N. clevelandii* in particular were highly susceptible to both CBSV and CBSUV in our conditions, and this could be an excellent differential host for separating severe and mild isolates. On *N. clevelandii*, the severe isolates CBSV-[MZ:Nam1-1:07] and CBSV-[TZ:Nal3-1:07] produced symptoms early, caused severe stunting of infected plants, leaf necrosis, and often plant death. The remaining isolates including CBSUV-[UG:Kab4-3:07] caused various forms of leaf chlorosis, the symptoms were less severe and nonlethal (Figure 2). To correlate the symptom diversity observed to the genetic diversity of CBSD isolates, the complete CP aa sequences of 24 isolates were compared with those reference sequences available in gene bank databases. Similar to the results obtained in previous studies [15, 16, 23], our virus isolates grouped into two clusters based on the two described species: CBSV and CBSUV (Figure 4). Based on aa sequence identities, the Ugandan epidemic isolate was highly similar to those from Kenya and Malawi, suggesting that CBSUV-[UG:Kab4-3:07] may have originated form one or both these countries.

Put together, these collective observations on symptom diversity as well as genetic differences did not indicate the association of a severe form of CBSD in Uganda. These results are indeed consistent with studies on another epidemic isolate (Namulonge) from Uganda [16] and especially agree with field observations in which the maximum average severity recorded at the onset of CBSD in Uganda was only 2.0 [5]. In the absence of a particularly virulent virus in Uganda, our results, however, raise serious questions as to the factors responsible for the current outbreaks of CBSD in eastern African countries. The possible explanations for this are the presence of unusually high populations of whitefly vectors (*B. tabaci*) on cassava that may be responsible for the rapid spread of the virus in the field. The recent widespread introduction of CMD-resistant varieties that are particularly susceptible to CBSD or the combination of both could be a factor in disease outbreak. Recent surveys in Uganda indeed confirmed these possibilities, where more than 70% of the cassavas grown in 23 districts were CMD-resistant improved varieties, all of which are susceptible to CBSD. These varieties also support high whitefly numbers, in excess of 200 adults per top five leaves. Although such elite cassava has not been introduced in high quantities to the Lake Zone Tanzania, the high susceptibility of local land races grown in the region and the sudden development of unusually high whitefly populations on cassava there is ensuring the spread of CBSD [6, 7]. Identification of severe forms of CBSVs in CBSD endemic regions is particularly worrying because the spread of these isolates into areas of high whitefly population has greater potential to cause even more severe damage to cassava production than yet encountered. Our results emphasize the need for exercising strict quarantine measures for preventing further spread of CBSD between country borders and have also identified the need for developing cassava varieties with broad spectrum resistance to both viruses.

## Figures and Tables

**Table 1:** List of CBSD isolates sequenced in this study; those used in the symptom diversity study are in bold.

Isolate name/abbreviation	Place and country of collection	Collection date	Accession number
CBSUV-[KE:Den1-2:08]	Denyenye, Kenya	October 2008	HM346937
CBSUV-[KE:Kil18-2:08]	Kilifi, Kenya	October 2008	HM346938
CBSUV-[KE:Kil20-1:08]	Kilifi, Kenya	October 2008	HM346939
CBSUV-[KE:Kil20-3:08]	Kilifi, Kenya	October 2008	HM346940
CBSUV-[KE:Dia3-1:08]	Diani, Kenya	October 2008	HM346941
CBSUV-[KE:Nyu5-4:08]	Nyumbasita, Kenya	October 2008	HM346942
CBSUV-[KE:Shi6-1:08]	Shirazi, Kenya	October 2008	HM346943
CBSUV-[KE:Shi7-1:08]	Shirazi, Kenya	October 2008	HM346944
CBSUV-[KE:Mri8-1:08]	Mrima, Kenya	October 2008	HM346945
CBSUV-[KE:Kik11-5:08]	Kikonde, Kenya	October 2008	HM346946
CBSUV-[KE:Kik10-1:08]	Kikonde, Kenya	October 2008	HM346947
CBSUV-[KE:Mba12-1:08]	Mwabandari, Kenya	October 2008	HM346948
**CBSUV-[KE:Mwa16-2:08]**	Mwalumba, Kenya	October 2008	HM346949
CBSUV-[KE:Chu21-1:08]	Chumani, Kenya	October 2008	HM346950
CBSUV-[KE:Nam2-1:08]	Namulonge, Uganda	December 2004	HM346951
**CBSUV-[UG:Kab4-3:07]**	Kabanyoro, Uganda	May 2007	HM346952
**CBSV-[MZ:Nam1-1:07]**	Nampula, Mozambique	November 2007	HM346953
**CBSV-[TZ:Nal3-1:07]**	Naliendele, Tanzania	November 2007	HM346954
**CBSUV-[TZ:Kib10-2:03]**	Kibaha, Tanzania	March 2003	HM346955
**CBSV-[TZ:Zan6-2:08]**	Zanzibar, Tanzania	October 2008	HM346956
CBSV-[TZ:Zan8-2:08]	Zanzibar, Tanzania	October 2008	HM346957
CBSV-[TZ:Zan7-1:08]	Zanzibar, Tanzania	October 2008	HM346958
CBSV-[TZ:Zan13-1:08]	Zanzibar, Tanzania	October 2008	HM346959
CBSV-[TZ:Zan11-1:08]	Zanzibar, Tanzania	October 2008	HM346960

**Table 2:** Herbaceous hosts inoculated with CBSD isolates.

Species/variety	Number of plants infected/ inoculated for each isolate						Total number of infected/ inoculated plants^1^ (%)
	CBSUV-			CBSV-			
	[UG:Kab4-3:07]	[KE:Mwa16-2:08]	[TZ:Kib10-2:03]	[TZ:Zan6-2:08]	[MZ:Nam1-1:07]	[TZ:Nal3-1:07]	
*Chenopodium quinoa *	0/10	0/10	0/10	0/10	0/10	0/10	0/60 (0.0)
*Cucurbita maxima*	0/10	0/10	0/10	0/10	0/10	0/10	0/60 (0.0)
*Datura metel*	0/10	0/10	0/10	0/10	0/10	0/10	0/60 (0.0)
*Datura stramonium*	4/10	2/10	2/10	3/10	9/10	4/10	24/60 (40.0)
*Solanum lycopersicum*	0/10	0/10	0/10	0/10	0/10	0/10	0/60 (0.0)
*Ipomoea batatas*	0/10	0/10	0/10	0/10	0/10	0/10	0/60 (0.0)
*Nicotiana benthamiana*	40/40	5/40	40/40	20/40	40/40	40/40	185/240 (77.0)
*Nicotiana clevelandii*	10/10	10/10	10/10	10/10	10/10	10/10	60/60 (100.0)
*Nicotiana glutinosa*	20/40	13/40	23/40	12/40	37/40	40/40	145/240 (60.0)
*Nicotiana hesperis*	0/10	0/10	0/10	0/10	0/10	0/10	0/60 (0.0)
*Nicotiana tabacum *nn	19/20	17/20	20/20	20/20	20/20	20/20	116/120 (96.6)
*Nicotiana tabacum* NN	10/10	10/10	10/10	7/10	9/10	10/10	56/60 (93.3)
*Nicotiana rustica*	18/20	17/20	15/20	20/20	20/20	20/20	110/120 (91.7)

Total number of infected/inoculated plants^2^ (%)	121/210 (57.6)	74/210 (35.2)	120/210 (57.1)	92/210 (43.8)	145/210 (69.0)	144/210 (68.6)	696/1260 (55.2)

^1^Number of infected plants for each cassava variety.

^2^Number of infected plants for each virus isolate.

**Table 3:** The rate of graft transmission of six CBSD isolates to different cassava varieties.

Cassava variety	Number of plants infected/grafted with each virus isolate						Total number of infected/grafted plants^1^ (%)
	CBSUV-			CBSV-			
	[UG:Kab4-3:07]	[KE:Mwa16-2:08]	[TZ:Kib10-2:03]	[TZ:Zan6-2:08]	[MZ:Nam1-1:07]	[TZ:Nal3-1:07]	
Albert	4/5	3/5	4/5	4/5	5/5	5/5	25/30 (83.3)
Kiroba	3/5	4/5	4/5	5/5	5/5	5/5	26/30 (86.6)
Ebwanateraka	3/5	4/5	3/5	3/5	5/5	5/5	23/30 (76.6)
Colombian	3/5	4/5	3/5	4/5	5/5	5/5	24/30 (80.0)
TMS 60444	2/5	4/5	3/5	4/5	5/5	5/5	23/30 (76.6)

Total number of infected/grafted plants^2^ (%)	15/25 (60.0)	19/25 (76.0)	17/25 (68.0)	20/25 (80.0)	25/25 (100)	25/25 (100)	121/150 (80.7)

^1^Number of infected plants for each cassava variety.

^2^Number of infected plants for each virus isolate.

**Table 4:** The effects of CBSD infections on the sprouting of cassava stem cuttings.

Cassava variety	Number of cuttings sprouted/planted when infected with each isolate^3^						Total number of sprouted/planted cuttings^1^ (%)
	CBSUV-			CBSV-			
	[UG:Kab4-3:07]	[KE:Mwa16-2:08]	[TZ:Kib10-2:03]	[TZ:Zan6-2:08]	[MZ:Nam1-1:07]	[TZ:Nal3-1:07]	
Albert	9/10	9/10	8/10	9/10	9/10	9/10	53/60 (88.3)
Kiroba	10/10	8/10	10/10	9/10	8/10	6/10	51/60 (85.0)
Ebwanateraka	10/10	7/10	10/10	8/10	9/10	10/10	54/60 (90.0)
Colombian	10/10	10/10	8/10	10/10	9/10	10/10	57/60 (95.0)
TMS 60444	9/10	10/10	10/10	5/10	4/10	2/10	40/60 (66.6)

Total number of sprouted/planted cuttings^2^ (%)	48/50 (96.0)	44/50 (88.0)	46/50 (92.0)	41/50 (82.0)	39/50 (78.0)	37/50 (74.0)	255/300 (85.0)

^1^Number of sprouted and fully grown plants for each cassava variety.

^2^Number of sprouted and fully grown plants for each virus isolate.

^3^All 10 cuttings were obtained from plants infected with viruses and showing typical CBSD symptoms.

**Table 5:** Mean symptom severity scores for each CBSD isolate on different cassava varieties (on a 0–5 scale using the procedure of [9]).

Cassava variety	Mean symptom severity scores for each virus isolate						Mean symptom severity^1^
	CBSUV-			CBSV-			
	[UG:Kab4-3:07]	[KE:Mwa16-2:08]	[TZ:Kib10-2:03]	[TZ:Zan6-2:08]	[MZ:Nam1-1:07]	[TZ:Nal3-1:07]	
Albert	1.9	2.9	2.2	2.8	4.0	3.9	3.0
Kiroba	1.9	2.0	2.0	2.4	3.0	2.7	2.3
Ebwanateraka	1.9	2.6	2.1	2.8	4.0	4.0	3.0
Colombian	1.9	2.9	2.1	2.9	4.0	4.0	3.0
TMS 60444	2.1	2.9	2.7	3.1	4.0	4.0	3.1

Mean symptom severity^2^	1.9	2.7	2.2	3.0	3.8	3.7	2.8

^1^Mean symptom severity for each variety.

^2^Mean symptom severity for each virus isolate.
